# Do Colorectal Serrated and Non-Serrated Adenocarcinomas Differ in Somatic Mutations and Clinicopathologic Features?

**DOI:** 10.3390/medicina61061032

**Published:** 2025-06-02

**Authors:** Zeynep Sagnak Yilmaz, Sibel Demir Kececi, Sevdegul Aydin Mungan, Ismail Saygin, Ozgul Sagol, Sulen Sarioglu

**Affiliations:** 1 Department of Molecular Pathology, Graduate School of Health Sciences, Dokuz Eylul University, 35340 Izmir, Turkey; sibeldemir2840@gmail.com (S.D.K.); ozgul.sagol@deu.edu.tr (O.S.); sulensari@gmail.com (S.S.); 2 Department of Pathology, Faculty of Medicine, Karadeniz Technical University, 61080 Trabzon, Turkey; drsevdegul@gmail.com (S.A.M.); ismail1974say@hotmail.com (I.S.); 3 Department of Pathology, Manisa City Hospital, 45040 Manisa, Turkey; 4 Department of Pathology, Faculty of Medicine, Dokuz Eylul University, 35340 Izmir, Turkey; 5 Memorial Health Group, Department of Pathology, 34381 Istanbul, Turkey

**Keywords:** colorectal carcinoma, serrated adenocarcinoma, non-serrated adenocarcinoma, somatic mutation, next-generation sequencing, clinicopathological features

## Abstract

*Background and Objectives*: Serrated adenocarcinoma (SAC) is a distinctive neoplasm that is histopathologically characterized by the presence of epithelial serration, an eosinophilic cytoplasm, and a vesicular nucleus. However, the literature data concerning somatic mutations in SACs remain extremely limited. *Materials and Methods*: A total of 159 colon resection cases diagnosed with adenocarcinoma whose DNA mutations were analyzed by next-generation sequencing (NGS) were retrospectively reviewed. In 23 cases, the SAC area exceeded 50%. A chi-square test was used to evaluate histopathologic characteristics and somatic mutations in SACs and non-serrated adenocarcinomas (non-SACs). *Results*: A significant difference was found in histological grade (*p* = 0.019) between SACs and non-SACs. *TP53*, *KRAS*, and *PIK3CA* genes have been identified as the most frequently mutated genes in both SACs and non-SACs. No statistically significant difference in somatic mutations was observed between the two groups (*p* > 0.05). *Conclusions*: In the present study, a higher prevalence of *KRAS* mutations was observed in SACs compared to *BRAF* mutations (*KRAS*: 39.1%, *BRAF*: 4.3%). This finding is consistent with the recent literature reporting a higher prevalence of *KRAS* mutations in colorectal SACs, in contrast to previous studies. The somatic mutation results of our study and the previous literature data suggest the potential importance of epigenetic alterations documented in the literature in the development of SACs.

## 1. Introduction

Serrated adenocarcinoma (SAC) is a World Health Organization (WHO) morphological variant of colorectal adenocarcinoma, which is characterized by the following: epithelial serration, an eosinophilic cytoplasm, a vesicular nucleus, the absence of necrosis and mucinous, or a trabecular growth pattern [1]. The term was first coined in 1992 by Jass and Smith, who reported five cases of colon adenocarcinoma that exhibited a resemblance to hyperplastic polyps [2]. According to the WHO 2010 classification system, it was designated as a distinct subtype of colorectal adenocarcinoma [3]. The progression of sessile serrated lesions (SSLs) and traditional serrated adenomas (TSAs) into carcinoma characterizes this transformation. However, it is noteworthy that most carcinomas arising from serrated precursors do not exhibit SAC morphology [1]. A histopathological examination reveals the presence of cell balls and papillary rods, which are characteristic findings. Necrosis is expected in less than 10% of tumor sections [4]. SACs constitute 5.8–12% of colorectal carcinoma (CRC). They are generally observed in the age range of 65–70 years. Most SACs are located in the right colon (47–57%) and rectum (15–29%) [5].

SAC patients demonstrate a higher incidence of lymph node metastasis (52%), and their prognosis is frequently less favorable in comparison to conventional adenocarcinomas [6]. Furthermore, SACs are distinguished by poor prognostic histological characteristics, including tumor budding and reduced peritumoral lymphocytic infiltration [5]. However, no definite conclusion has been reached regarding the prognosis of SACs. Some studies have reported that it is a subtype of CRC with a poor prognosis [7,8]; in contrast, Mäkinen et al. [9] reported no significant difference in cancer-related mortality between SACs and conventional adenocarcinoma.

A review of the extant literature reveals that studies comparing SACs with conventional adenocarcinoma are generally based on gene expression and methylation molecular studies. Genes related to morphogenesis, the cytoskeleton, and hypoxia were associated with SACs. HIF-1alpha, fascin 1, the anti-apoptotic gene hippocalcin, and annexin A10 were upregulated in SACs. It is possible to use these biomarkers as immunohistochemical (IHC) markers [10,11].

Molecularly, mutations in the *BRAF* and *KRAS* genes are essential, particularly in the serrated pathway. The *BRAF* and *KRAS* genes encode kinases belonging to the mitogen-activated protein kinase (MAPK) cascade. These cascades are involved in the cell signaling that drives cell proliferation and differentiation. Mutations in the *KRAS* and *BRAF* oncogenes promote invasion and metastasis by activating the MAPK pathway, cell proliferation, and cell survival [12]. Most cases are derived from the TSAs and are characterized as microsatellite stable (MSS) or microsatellite instability-low (MSI-L). A minority of cases are caused by SSL, which is usually associated with high microsatellite instability (MSI-H) [13]. A study in the literature reported that mutations in *KRAS* genes were more common than *BRAF* genes in SACs. [14]. However, another study stated that *BRAF* mutations were significantly higher than *KRAS* [6]. As an epigenetic alteration, CIMP is associated with the serrated pathway. In serrated pathway-associated lesions, CIMP-H is related to a *KRAS* mutation, and CIMP-L is related to a *BRAF* mutation [15]. It was observed that ½–⅓ of the CRCs developing from the serrated pathway showed serrated morphology and were grouped as SAC. The remaining cases were conventional CRC cases that developed from the serrated pathway, but did not demonstrate serrated morphology. They were more commonly known to show the CpG Island Methylator Phenotype-Low (CIMP-L) [16]. In methylation studies, DIO3 and FOXD2 exhibited higher methylation levels and lower mRNA expression in SACs than in CRCs [15]. The studies of RNA molecular structures in SACs have been performed on microRNA rather than fusion. An increased expression of microRNA 31 has been demonstrated to play a significant role in the serrated pathway that leads to colon cancer [17,18].

We aimed to compare this subtype characterized by serrated morphology with CRCs without serrated morphology in terms of clinicopathological parameters and molecular alterations. Although previous studies have focused on cell morphogenesis and cytoskeleton-related genes, we noticed that there is limited data in the literature on the somatic mutations associated with SAC. To further understand this serrated morphology variant of CRC, we report our experience in analyzing our available clinicopathological and molecular data.

## 2. Material and Methods

### 2.1. Materials

**Figure 1:**
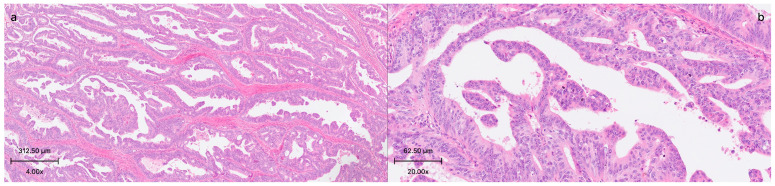
(**a**,**b**): Serrated adenocarcinoma. Low (**a**) and intermediate (**b**) view of serrated adenocarcinoma composed of epithelial serrations, eosinophilic cytoplasm, and papillary rods (Hematoxylin and eosin).

A total of 159 adenocarcinoma colon resection samples that underwent somatic DNA and RNA mutations by next generation sequencing (NGS) analysis between 2019 and 2023 were re-examined. A retrospective examination of the cases was conducted for the presence of serrated carcinoma morphology, relying on histopathological analysis. Cases with the morphological characteristics of SAC were defined using Makinen’s criteria, such as epithelial serrations, eosinophilic or clear cytoplasm, abundant cytoplasm, vesicular nuclei, distinct nucleoli, preserved polarity, necrosis in less than 10% of the total area, or the absence of necrosis and the presence of cell balls and papillary rods within mucinous areas. Those which showed at least 6 of these 7 features were grouped as SAC (Figure 1a,b). Papillary projections with fibrovascular cores and serrated-like structures resulting from tumor cell necrosis were excluded [19]. As stated in the literature, cases in which at least 50 percent of tumor samples showed this feature were accepted as SAC [20]. The histology was confirmed by two independent pathologists (S.Y.Z. and S.S.) from the hematoxylin and eosin-stained slides.

### 2.2. Histological and Clinical Findings

The anatomical location of the neoplasm, histopathological grade, tumor size and stage (T and N), lymphovascular invasion, perineural invasion, distant organ metastasis, and demographic data (gender and age) were obtained from the patients’ medical records.

### 2.3. DNA/RNA Extraction

The isolation of DNA and RNA from the paraffin-embedded tumor tissues of the cases was conducted using the QIAGEN GeneRead DNA/RNA Formalin-Fixed and Paraffin-Embedded (FFPE) Kit (Qiagen, Hilden, Germany) The quantity of the DNA/RNA obtained (Qubit 4) was sufficient to complete this study. The quality of the extracted DNA/RNA was determined by means of the gel run method (Qiaxcel, Qiagen, Hilden, Germany).

### 2.4. Next Generating Sequencing

The investigation of somatic mutations was conducted using NGS technology (Ilumina MiSeq platform). A variety of NGS DNA panels were utilized in this study, including the QIAsec new large solid custom microsatellite instability (MSI) panel (63 cases), the QIAsec new solid custom MSI panel (38 cases), the QIAsec solid tumor custom panel (40 cases), the QIAact AIT DNA UMI panel (16 cases), and the AIT Basic QIAact Actionable Insights Tumor Panel (2 cases) (Qiagen, Hilden, Germany). It was observed that the genes evaluated differed in some cases. The reason for applying different panels to the cases was that this study was retrospective and NGS was previously performed in these cases with the panels previously used in routine practice in a molecular laboratory (panels were updated from past to present). Therefore, the same gene panel was not used in every case in the past and the gene list evaluated per case varied.

The cases analyzed for specific exons for DNA somatic mutations by NGS were as follows: the *KRAS*, *NRAS*, *PIK3CA*, *BRAF*, *KIT*, *EGFR*, and *ERBB2* genes in 159 cases; the *HRAS*, *BRCA2*, *IDH1*, and *ROS1* genes in 141 cases; the *PTEN* gene in 142 cases; the *TP53* gene in 117 cases; the *TERT* gene in 118 cases; the *POLE* gene in 66 cases; the *BCOR* gene in 31 cases; the *FGFR1*, *CTNNB1*, and *SMAD4* genes in 15 cases; the *ERBB3* gene in 18 cases; the *MAP2K1* gene in 16 cases; and the *FBXW7* gene in 14 cases.

The RNA isolation process was executed in 19 cases employing the QIAsec RNA large fusion panel (CFHS-11888Z-165). The presence of RNA fusions was investigated in in specific exons of *ALK*, *BAIAP2L1*, *BRAF*, *BRD4*, *CEP89*, *CLCN6*, *CLIP4*, *EGFR*, *EML4*, *ETV6*, *FGFR1*, *FGFR2*, *FGFR3*, *FN1*, *GATM*, *GNAI1*, *HIP1*, *KAP9*, *KIF5B*, *KLC1*, *LMNA*, *MET*, *NACC2*, *NPM1*, *NRG1*, *NTRK1*, *NTRK2*, *NTRK3*, *PLAG1*, *QKI*, *RANBP2*, *RET*, *ROS1*, *SEC31A*, *SLC45A3*, *SND1*, *TACC1*, *TCF3*, *TFG*, *TPM3*, *TPM4*, *TPR*, *TPR*, *VCL*, *WDCP*, and *ZNF703* by NGS. Genomic alterations characterized by exon skipping in the *MET* and *EGFR* genes were also examined by this RNA panel.

The analysis of microsatellite instability (MSI) by an examination of specific loci, including BAT40(T)37, MONO-27(T)27, BAT26(A)27, NR24(T)23, BAT25(T)25, NR22(T)21, HSP110-T17(T)17, NR21(A)21, and BAT34C4(A)18, was performed in 106 cases by NGS. The assessment of MSI status is determined using the following cut-off ranges: MSI-H > 40% instability, MSI-L 15–40% instability, and MSS < 15% instability.

### 2.5. Variant Detection

NGS was subsequently performed on the Illumina NovaSeq system. Bioinformatic analysis was performed using the Qiagen Clinical Insight Interpret and CLC Genomic Workbench interfaces. Variants were detected if they had a reading depth of 500×, an Allele Frequency of 5% or greater, and a QUAL score of 200 or greater. The variants identified were categorized according to Tier Classification. Clinically significant variants were classified as pathogenic or likely pathogenic variants. Pathogenic and likely pathogenic variants were included, and variants of uncertain significance (VUS), likely benign and benign variants, were not included in this study.

### 2.6. Statistical Analysis

The relationship between histopathologic parameters, somatic DNA mutations, RNA fusions, and MSI status between SACs and non-SACs was statistically evaluated. The statistical analysis program SPSS 29.0 was used. Statistical significance between two independent groups was determined by Student’s *t*-test if normal distribution was provided. The variables between two groups were compared by chi-squared test. A *p*-value < 0.05 was considered statistically significant.

### 2.7. In Silico Analysis

Somatic mutations detected in our SAC cases were analyzed using in silico methods (g:Profiler, a public bioinformatics database) to identify associated pathways [21]. Additionally, the in silico evaluation of the frequency of *ROS1* and *RET* RNA fusion mutations in patients with CRC was conducted by using the Colorectal Cancer (MSK, JNC 2021) dataset from the cBioPortal for Cancer Genomics public database [22,23,24,25] This was undertaken due to the unavailability of literature on the subject.

## 3. Results

### 3.1. Clinicopathological Characteristics

Serrated morphology was detected in 23 cases (14.5%), and 136 cases (85.5%) showed no serrated features. The clinicopathological features are listed in Table 1. No significant difference was found between the SAC and non-SAC groups in terms of age, gender, and location. Histological grades were significantly different between the SAC and non-SAC groups (*p* = 0.019) (Table 1).

The analysis revealed that both SACs and non-SACs were more frequently observed in men (SAC: 73.9%, non-SACs: 66.2%). The mean age was 62.5 for the SAC group and 61.6 for the non-SAC group. A substantial proportion of cases were located in the sigmoid colon and rectum, accounting for 59.4% of all cases. SACs were predominantly located in the sigmoid colon and rectum (27.8% each), followed by the ascending and descending colon (11.1% each). Non-SACs showed a similar distribution, with the sigmoid colon and rectum being the most common sites (30% each). This was followed by the ascending colon (13.3%).

The majority of SAC and non-SAC cases were grade 2 (95.7% and 71.3%, respectively). Histologically, 95.7% of SAC cases were grade 2 and 4.3% were grade 1. In contrast, non-SACs showed a dissimilar distribution, with grade 2 (71.3%) being the most common, followed by grade 3 (20.6%) and grade 1 (8.1%). A statistically significant difference was observed between the histologic grades of SACs and non-SACs (*p =* 0.019). The most prevalent T stage in both SACs and non-SACs was T3 (SACs: 78.9%; non-SACs: 68.9). Both groups’ second most prevalent stage was T4 (SACs: 21%; non-SACs: 37.9%). Notably, none of the SACs exhibited T1 or T2 stages.

Lymph node metastasis was observed in 12 SAC cases (63.2%). In contrast, among the non-SAC cases, lymph node metastasis was observed in 73.4% of cases. However, there was no statistically significant difference (*p* > 0.05). Tumor deposits were detected at a higher rate in the non-SAC cells compared to the SACs (52.3% vs. 35.7%). Additionally, lymphovascular invasion was found to be more prevalent in SACs compared to non-SACs (60.9% vs. 56.6%). The presence of perineural invasion was similar in both groups.

In the present study, cases with information about distant metastasis were 12/12 of the SAC cases and 95/103 of the non-SAC cases. The liver was the most frequently involved organ in seven SACs. One case had peritoneal metastasis, and the other cases had metastasis to distant organs. Similarly, the most common metastatic organs were the liver (41 cases) and peritoneum (17 cases) in the non-SAC cases. No statistically significant difference was found between the two groups in terms of metastatic sites.

### 3.2. Mutations and Microsatellite Instability

Table 2 presents all somatic mutations observed in the SAC and non-SAC cases. The most common somatic mutations in all cases were *TP53* (66.7%, 78/117), *KRAS* (44%, 70/159), and *PIK3CA* (21.4%, 34/159). The percentage of *KRAS* mutations in non-SACs was higher than in SACs (non-SACs: 44.9%, 61/136, SACs: 39.1%, 9/23), but the difference was not statistically significant (*p* > 0.05). The *TP53* mutation was determined in 61.1% of SACs (11/18 cases) and in 67.7% of non-SACs (67/99 cases). The *PIK3CA* mutations were more frequently observed in SACs (6/23, 26.1%) compared to non-SACs (20.6%, 28/136). There was no statistically significant difference in all these genes between the two groups. Somatic mutations were detected in the *BRAF*, *NRAS* and *KIT* genes in one case each in the SAC group. The other somatic gene mutations in the non-SACs are presented in Table 2.

In 11 of the SACs, MSI was evaluated by NGS; 9 were MSS (81.8%) and 2 were MSI-L (18.2%). No MSI-H was detected in the SACs. Of the 80 non-SAC cases, 63 (78.8%) were classified as MSS, 8 (10%) as MSI-L, and 9 (11.3%) as MSI-H. Statistically, no significant difference was detected between the SACs and non-SACs in terms of MSI and somatic mutations (Table 2).

An RNA-Fusion analysis was performed in 2 of the SAC cases and 17 of the non-SAC cases. No fusion was detected in the SACs while one non-SAC case showed RET-ASCCE fusion and two non-SAC cases showed ROS1 fusions (ROS1-ANKH in one case and ROS1-KLHL25 in another).

### 3.3. In Silico Analysis

**Figure 2:**
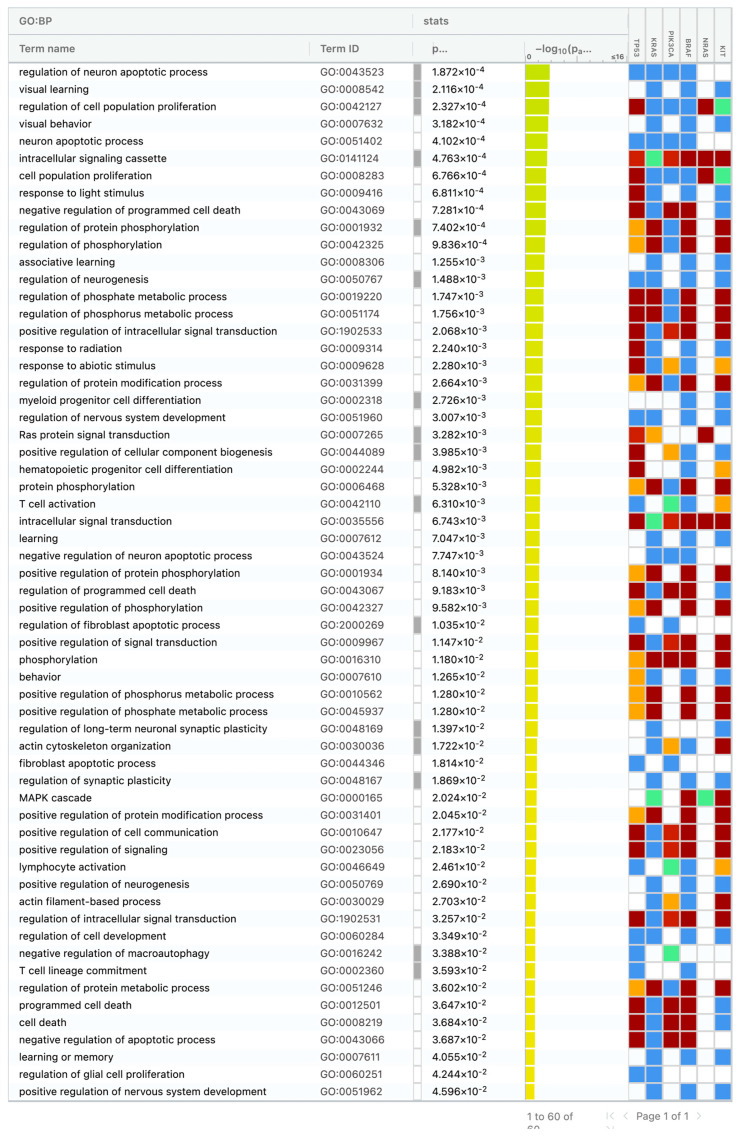
Gene pathways associated with expressed somatic DNA mutations in serrated adenocarcinomas from g:Profiler.

The following pathways have been associated with mutated genes in SACs (*TP53*, *KRAS*, *PIK3CA*, *BRAF*, *NRAS*, *KIT*), in order of frequency, the regulation of the neuron apoptotic process, visual learning, the regulation of cell population proliferation, visual behavior, the neuron apoptotic process, the intracellular signaling cassette, the response to light stimulus, cell population proliferation, and others (Figure 2) [21].

We investigated the *ROS1* and *RET* fusions concerning mutation rates in the CRC (MSK, JNC 2021) study from the cBioPortal for cancer genomics database. In this study, 1516 samples of CRC were sequenced. ROS1-GOPC fusion was detected in one case (0.06%) and RET-NCOA4 fusion was detected in one case in this study (0.06%) [22,23,24,25].

## 4. Discussion

The prevalence of SACs among CRCs ranges from 5.8% to 12% in the literature [5]. In this study, SACs constituted 14.5% (n = 23) of our cases, which was slightly higher than in the literature data. Studies on gender predominance in SACs and non-SACs had yielded conflicting results. Our research found that SACs were more common in men (73.9%), and this finding is consistent with the literature data [7,19,26]. A review of the relevant literature reveals that the mean age of SAC and non-SAC patients was over 65 years. In our study, the mean age was younger (SACs: 62.5, non-SACs: 61.6) than the values reported in the literature. The mean age is generally higher in non-SAC patients, but in one study, it was higher in SAC patients [6,8,10,19,27]. In the present study, the mean age in SAC patients was higher than in non-SAC patients.

A review of the current literature reveals that, according to the location of SACs, the distal colon/rectum was the most common localization in some studies, while the proximal colon was the most common localization in other CRCs [6,7,8,10,19,27]. In our present study, the most prevalent location of SACs was the sigmoid colon and rectum. Our study concluded that SACs were seen in the proximal colon in a lower percentage compared to the literature data. Conversely, non-SACs exhibited a predilection for distal colon localization in our study and this finding is consistent with the recent literature [6,7,8,19,26]. Among the precursor lesions, TSA was characteristically located in the distal colon and rectum, while SSL was located in the proximal colon [28]. The higher frequency of SAC cases in the distal colon and rectum, as observed in this study and some cases documented in the literature, suggests that these cases most likely developed from TSA.

In the research conducted, SACs and non-SACs were found to be mostly moderately differentiated (grade 2) [6,7,8]. Furthermore, the percentage of grade 2 cases in SACs was found to be higher in our study (95.7%) compared to the literature data. In accordance with the findings of Shida et al. [7], no grade 3 SACs were detected in our study. Our findings showed that SACs were better differentiated than non-SACs, and there was a significant difference (*p* = 0.019). As glandular structures with serrated morphology have not yet lost their differentiation, poorly differentiated or high-grade components are not expected to be common in SACs.

A literature review showed that the T stages were T3 and T4 in SACs and non-SACs, as seen in our study [8,26]. Our study showed that T3 and T4 were the most common stages in both groups, though there were no significant differences between the groups. None of the SACs showed stages T1 or T2. This may be related to the following feature of our case series. All cases included in this study required targeted therapy, which necessitated the examination of molecular changes. Consequently, the cases in our study were generally at an advanced stage and metastatic.

Studies show that 43.3–53.9% of SACs have lymph node metastasis [7,10,19,26]. In the current study, a higher rate of lymph node metastasis was observed in SACs (63.2%) compared to the literature data. Lymph node metastasis was found to be more common in SACs than in non-serrated CRCs [10,19,27]. In our study, higher rates of lymph node metastasis and tumor deposits were observed in non-SACs compared to SACs. A total of 73.4% of non-SACs exhibited lymph node metastasis, while 63.2% of SACs did. Moreover, a higher prevalence of tumor deposits was observed in non-SACs in comparison to SACs (non-SACs: 52.3%, SACs: 35.7%). This may be related to the fact that the patients in our case series were in an advanced stage and required targeted therapy. Additionally, similar rates of perineural invasion were observed in both groups (SACs: 30.4% vs. non-SACs: 30.9%). There is not sufficient research on perineural invasion and tumor deposits in SACs and further research is needed in the literature. Lymphovascular invasion was found in a variable range of 16.6–81.8% in SACs, and no significant difference was found between SACs and non-SACs in the literature data [7,26]. In our study, lymphovascular invasion was detected more frequently in SACs than in non-SACs (60.9% vs. 56.6%), and the *p* value was >0.05, as reported in the literature.

The research on distant organ metastasis in SACs showed a range of demonstrated prevalence from 14.4% to 25% in the literature. While this range exceeds the rate observed in other CRCs, no significant difference was found [6,8,26]. In contrast, our study showed metastasis in all cases of SACs (100%), while metastasis was documented in 92.2% of non-SACs. These rates were very high compared to the literature data. Our study population comprised patients requiring targeted therapy who underwent a molecular analysis of tumor tissue. Therefore, the majority of cases were metastatic.

The literature data on somatic mutations in colorectal SACs is limited. We observed *TP53*, *KRAS*, and *PIK3CA* mutations most frequently in both SACs and non-SACs, and we found no statistically significant difference between the two groups (*p* < 0.05). In the current study, somatic mutations in the *TP53* gene were examined in 66.1% SACs and 67.7% non-SACs. Given the fact that a *TP53* mutation is the most prevalent somatic mutation and is associated with advanced stage and poor prognosis, the high rate of *TP53* mutations observed in both groups in our case series is not unexpected [29]. In a related study by Hirano et al. [12], 24 early-stage SAC cases were examined for mutations, and *TP53* mutations were identified in 66.6% of cases. This rate is consistent with the findings of our study.

The literature indicates that the rate of *KRAS* mutation in SACs varies between 42.7% and 58.3%, and that it has been found more frequently in SACs compared to conventional CRCs [6,12,27]. However, in our study, we observed a slightly lower prevalence of *KRAS* mutations in SACs (39.1%) compared to the literature data, thereby offering an alternative perspective on this phenomenon. Additionally, the *KRAS* mutation rate was found to be slightly lower in SACs when compared to non-SACs (SACs: 39.1%; non-SACs: 44.9%). The prevalence of *BRAF* mutations varies in the existing literature (16.6–33%) [6,12,20,27]. Our study observed a *BRAF* mutation in a single SAC case (4.3%). In non-SACs, a *BRAF* mutation was detected in 8.1% of cases. According to the literature, a *BRAF* mutation was detected at a low rate in the SACs in our study.

The study by Stefanius et al. [6]. demonstrates that *KRAS* mutations were present in 45.2% of SACs, and that MAPK activation caused by *KRAS* or *BRAF* mutations was very common in SACs (78.5%). Previous studies stated that *BRAF* exhibits biological predominance over *KRAS* within the serrated pathway. It was previously thought that a *KRAS* mutation was specific only to CRCs developing from Vogelstein’s classical adenoma–carcinoma sequence [14,30]. A significant proportion of *KRAS*-mutated CRCs have been observed to originate from serrated lesions [31]. After the terms ‘serrated pathway’ and ‘SAC’ were defined, more *KRAS* mutations were found in these neoplasms. This shows that a *KRAS* mutation is a more significant alteration in the serrated pathway than previous studies indicated.

Furthermore, the prevalence of *BRAF* and *KRAS* mutations differs in TSAs and SSLs. A *KRAS* mutation has been identified as the predominant genetic abnormality in TSAs (80% of cases). The prevalence of *BRAF* mutations in SSLs has been documented to range from 75% to 82% [6]. Considering the occurrence of both SACs and non-SACs in the rectum and left colon in our cases, it is reasonable to hypothesize that most cases may have arisen from TSA. This may explain the higher incidence of *KRAS* mutations compared to *BRAF* mutations in our study. The study by Stefanius et al. [6] and Solano et al. [27] showed a higher frequency of *KRAS* mutations in SACs compared to conventional CRCs. This suggests that a *KRAS* mutation may be a more significant alteration in the serrated pathway than a *BRAF* mutation. This finding is not consistent with the consensus that *BRAF* mutations are a hallmark of SAC development. The present study lends further support to this theory, through its observation of a significantly higher prevalence of *KRAS* mutations compared to *BRAF* mutations in SAC cases.

The *PIK3CA* mutation was identified in 9–16.6% of SACs in the literature [12,27]. These percentages were lower than the percentage of *PIK3CA* mutations in SACs in our study (26.1%). In the study by Solano et al. [27], a *PIK3CA* mutation was found to be more frequent in SACs than in non-SACs, as also observed in the present study. In our study, we also identified *NRAS* and *KIT* mutations in a single SAC case each (4.3% of SAC cases), while a *KIT* mutation was not detected in non-SACs and an *NRAS* mutation was detected in 3.7% of cases in this group. In the study by Hirano et al. [12], both *NRAS* and *KIT* mutations were detected in 29.1% of SACs. However, no data set in the literature directly compare SACs and non-SACs concerning the prevalence of these mutations. A *KIT* mutation was not observed in non-SAC cases; however, a *KIT* mutation was detected in one of 23 SAC cases, suggesting its rarity in this specific pathway.

Our study revealed that MSI-H was not detected in SACs while it was detected in 11.3% of non-SACs. The serrated pathway comprises two distinct, parallel pathways that contribute to tumor pathogenesis. One of these pathways is distinguished by proximal colon localization, *BRAF* mutations, *MLH1* silencing, and MSI-H, while the other pathway is characterized by distal colon/rectal localization, *KRAS* mutations, and MSS [32]. It has been observed that MSI-H is associated with *BRAF*-mutated cases. The literature review showed that the incidence of MSI-H in SACs ranged from 9.1% to 20.8% and that these MSI-H cases were associated with the occurrence of *BRAF* mutations [6,12,20,27]. Since the majority of cases in our case series were *KRAS* mutated, the MSS/MSI-L status of SACs was an expected result of our study.

In our study, the number of cases evaluated for RNA fusion was limited. Therefore, it is not possible to make meaningful comparisons between the two groups. According to the cBioPortal database, *ROS1* and *RET* fusion were present in only one case each (0.06%) in CRCs [22,23,24,25]. Conversely, our study did not detect fusion in two SAC cases for which an RNA-NGS analysis was performed. The literature contains no data on the fusion mutations observed in SACs, highlighting the necessity for further study in this area. An analysis of the pathways associated with somatic mutations obtained in our study using g:Profiler revealed the involvement of numerous pathways, including those associated with the neuron apoptotic process, cell proliferation, and intracellular signaling. The pathway analysis did not yield specific results for the SACs associated with somatic mutations. The literature data suggest that SACs may be related to epigenetic mechanisms rather than somatic mutations.

In the previous studies on SACs, it was determined that this form of CRC was more advanced and associated with a worse prognosis than conventional adenocarcinomas [19]. Several studies have indicated that the SAC subtype may exhibit a more aggressive course. However, other studies have suggested that aggressiveness may depend on MMR status, precursor polyp subtype, and tumor location [8,9,13,19]. In the study by Lee et al., mucinous tumors with serrated morphology were found to be associated with increased survival when they exhibited serrated morphology [33]. In a recent study by Yılmaz et al., an improvement in survival was found in SACs compared to non-SACs [20]. Further studies are necessary to reach a definitive conclusion regarding the prognosis of SACs.

A proportion of carcinomas arising from the serrated pathway progress to conventional CRC. This observation may offer a potential explanation for the variability in molecular differences between the SACs and conventional CRCs observed in different studies. This could also explain why we did not observe significant differences between the groups in our study. Given the potential for CIMP-L CRCs to originate from both the classical pathway and the serrated neoplasia pathway, there is a compelling need to develop molecular markers that can effectively identify CIMP-L SACs. Further studies are needed to fully understand the clinical impact of a SAC diagnosis. The identification of mutations that may prove effective in the formation of this subtype of colon adenocarcinoma, which has different biological characteristics, may also be useful for the development of targeted therapy.

## 5. Conclusions

Our study suggests that *KRAS* mutations may be more significant than *BRAF* mutations for SACs. It should be kept in mind that mutations in SAC may vary depending on the precursor polyp subtype, tumor location, and MMR status. However, the absence of a unifying molecular signature for the serrated pathway underlines the need for further investigation to elucidate the relationship between the serrated cancer pathway and SAC. Further studies are necessary to determine whether SAC is a distinct biological entity or merely a subtype of CRC. This study, when considered together with the existing data, suggests that epigenetic changes may play a more critical role in the development of SACs than somatic mutations.

## 6. Limitations

In this retrospective study, gene analysis could not be performed uniformly among all cases. Most cases included in this study were advanced in stage and metastatic. The evaluation of epigenetic data was not a component of this study; however, the necessity for such evaluations is paramount in this field. Further research in the form of epigenetic studies is therefore recommended.

## Figures and Tables

**Table 1:** The clinical and pathological features of serrated and non-serrated adenocarcinomas.

	Serrated Adenocarcinomas	Non-Serrated Adenocarcinomas	*p*
Gender			0.65
Male	73.9% (17/23)	66.2% (90/136)	
Female	26.1% (6/23)	33.8% (46/136)	
Age			0.72
Mean ± SD *	62.5 ± 15.4	61.6 ± 11.0	
Location			0.15
Sigmoid colon	27.8% (5/18)	30% (36/120)	
Rectum	27.8% (5/18)	30% (36/120)	
Ascending colon	11.1% (2/18)	13.3% (16/120)	
Descending colon	11.1% (2/18)	8.3% (10/120)	
Transverse colon	22.2% (4/18)	3.3% (4/120)	
Splenic flexura	0	3.3% (4/120)	
Hepatic flexura	0	2.5% (3/120)	
Cecum	0	9.2% (11/120)	
Histological Grade			0.019
Grade 1	4.3% (1/23)	8.1% (11/136)	
Grade 2	95.7% (22/23)	71.3% (97/136)	
Grade 3	0	20.6% (28/136)	
pT stage			0.49
pT1	0	1.6% (2/124)	
pT2	0	1.6% (2/124)	
pT3	78.9% (15/19)	68.9% (73/124)	
pT4a	10.5% (2/19)	27.4% (34/124)	
pT4b	10.5% (2/19)	10.5% (13/124)	
pN stage			0.08
pN0	36.8% (7/19)	26.6% (33/124)	
pN1a	5.3% (1/19)	14.5% (18/124)	
pN1b	10.5% (2/19)	19.4% (24/124)	
pN1c	15.8% (3/19)	2.4% (3/124)	
pN2a	15.8% (3/19)	14.5% (18/124)	
pN2b	15.8% (3/19)	22.6% (28/124)	
Tumor deposits			0.25
Present	35.7% (5/14)	52.3% (46/88)	
Absent	64.3% (9/14)	47.7% (42/88)	
Lymphovascular invasion			0.70
Present	60.9% (14/23)	56.6% (77/136)	
Absent	39.1% (9/23)	44.3% (59/136)	
Perineural invasion			0.96
Present	30.4% (7/23)	30.9% (42/136)	
Absent	69.6% (16/23)	69.1% (94/136)	
Metastasis (pM)			0.32
Present	100% (12/12)	92.2% (95/103)	
Absent	0	7.8% (8/103)	

* SD: standard deviation.

**Table 2:** Somatic DNA mutations and microsatellite instability in serrated and non-serrated adenocarcinomas.

	Serrated Adenocarcinomas	Non-Serrated Adenocarcinomas	*p*
*TP53*	61.1% (11/18)	67.7% (67/99)	0.58
*KRAS*	39.1% (9/23)	44.9% (61/136)	0.60
*PIK3CA*	26.1% (6/23)	20.6% (28/136)	0.58
*BRAF*	4.3% (1/23)	8.1% (11/136)	>0.99
*NRAS*	4.3% (1/23)	3.7% (5/136)	>0.99
*KIT*	4.3% (1/23)	0 (0/136)	0.14
*FBXW7*	0 (0/1)	15.4% (2/13)	>0.99
*SMAD4*	0 (0/1)	7.1% (1/14)	>0.99
*FGFR1*	0 (0/1)	7.1% (1/14)	>0.99
*CTNNB1*	0 (0/1)	7.1% (1/14)	>0.99
*ERBB3*	0 (0/3)	6.7% (1/15)	>0.99
*MAP2K1*	0 (0/1)	6.7% (1/15)	>0.99
*ERBB2*	0 (0/23)	4.4% (6/136)	0.59
*BCOR*	0 (0/5)	3.8% (1/26)	>0.99
*POLE*	0 (0/10)	3.6% (2/56)	>0.99
*PTEN*	0 (0/20)	3.3% (4/122)	>0.99
*BRCA2*	0 (0/20)	2.5% (3/121)	>0.99
*TERT*	0 (0/16)	1% (1/102)	>0.99
*IDH1*	0 (0/20)	0.8% (1/121)	>0.99
*ROS1*	0 (0/20)	0.8% (1/121)	>0.99
*HRAS*	0 (0/20)	0.8% (1/121)	>0.99
*EGFR*	0 (0/23)	0.7% (1/136)	>0.99
Microsatellite instability	MSS—81.8% (9/11)	MSS—78.8% (63/80)	0.399
	MSI-L—18.2% (2/11)	MSI-L—10% (8/80)	
	MSI-H—0 (0/11)	MSI-H—11.3% (9/80)	

Microsatellite instability Stable: MSS; Microsatellite instability-Low: MSI-L; Microsatellite instability-High: MSI-H. Bold marked genes are mutant in serrated colorectal carcinomas.

## Data Availability

Data are contained within the article. The original contributions presented in this study are included in the article. Further inquiries can be directed at the corresponding author.

## Abbreviations

CRC	Colorectal cancer
CIMP-H	Cpg Island Methylator Phenotype-High
CIMP-L	CpG Island Methylator Phenotype-Low
FFPE	Formalin-Fixed and Paraffin-Embedded
IHC	Immunohistochemical
MAPK	Mitogen-activated protein kinase
MSI	Microsatellite instability
MSI-L	Microsatellite instability-low
MSI-H	Microsatellite instability-high
MSS	Microsatellite stable
NGS	Next-generation sequencing
non-SACs	Non-serrated adenocarcinomas
SAC	Serrated adenocarcinoma
SSL	Sessile serrated lesion
TSA	Traditional serrated adenoma
WHO	World Health Organization
